# Burden of RSV-Associated Inpatient Care and Emergency Service Utilization in Two German Pediatric Centers Across Six Seasons Including the First Nirsevimab Year

**DOI:** 10.3390/children13020173

**Published:** 2026-01-26

**Authors:** Lisa Gürke, Gregor Hanslik, Linda-Marie Mulzer, Stefan Zimmermann, Heiko Reutter, Patrick Morhart, Joachim Wölfle, Michael K. Baumgartner, Anna-Lena Behr, Anne Christina Garbe, Hans-Christoph von Andrian, Melanie L. Conrad, Fabian B. Fahlbusch, Steven Hébert

**Affiliations:** 1 Division of Neonatology and Pediatric Intensive Care Medicine, Department of Pediatrics and Adolescent Medicine, Friedrich-Alexander-Universität of Erlangen-Nürnberg, 91054 Erlangen, Germany; 2 Department of Pediatrics, Medical Campus Oberfranken, Friedrich-Alexander-Universität of Erlangen-Nürnberg, 95445 Bayreuth, Germany; 3 Department of Pediatrics and Adolescent Medicine, Friedrich-Alexander-Universität of Erlangen-Nürnberg, 91054 Erlangen, Germany; 4 Neonatology and Pediatric Intensive Care, Department of Pediatrics and Adolescent Medicine, Faculty of Medicine, University of Augsburg, 86156 Augsburg, Germany

**Keywords:** respiratory syncytial virus (RSV), pediatric intensive care unit (PICU) burden, emergency department (ED) utilization, infant immunoprophylaxis (Nirsevimab), severe pediatric respiratory infection

## Abstract

**Highlights:**

**What are the main findings?**

- RSV caused sustained emergency department and inpatient utilization across six seasons.
- Overall severity remained stable; severe RSV disease (PICU admission or invasive respiratory support) occurred only in unimmunized infants during the first Nirsevimab season.

**What are the implications of the main findings?**

- RSV continues to impose a relevant outpatient and ward-level healthcare burden despite stable population-level severity.
- Integrated inpatient and emergency department data enable nuanced assessment of preventive strategies beyond incidence alone.

**Abstract:**

**Background/Objectives:** Respiratory syncytial virus (RSV) is a major cause of infant respiratory morbidity, yet real-world data on healthcare utilization following universal immunoprophylaxis remain limited. **Methods:** We retrospectively analyzed RSV-positive inpatient and emergency department (ED) encounters from two German tertiary pediatric centers across six seasons (2019/20–2024/25). Augsburg contributed inpatient data for 2022/23–2024/25, including immunization status and severity metrics, while Erlangen provided inpatient and ED data across all seasons. **Results:** In Augsburg, RSV hospitalizations were higher in pre-immunization seasons than in 2024/25, accompanied by a reduced proportion of infants < 1 year. RSV season onset occurred later in 2024/25, while severity metrics remained stable. Among infants < 1 year hospitalized in 2024/25, all severe cases occurred in unimmunized infants; no severe outcomes were observed among the small number of immunized cases. **Conclusions:** Integrated multicenter data descriptively coincide with reduced RSV hospitalization burden following immunoprophylaxis introduction, without evidence of increased disease severity.

## 1. Introduction

Respiratory syncytial virus (RSV) is a leading global cause of acute lower respiratory tract infection in infants and young children, resulting in substantial morbidity and mortality and placing a considerable burden on pediatric emergency and intensive care services worldwide [1,2,3]. In 2019, RSV was responsible for an estimated 33 million lower respiratory tract infections and 3.6 million hospitalizations among children under five years of age, with the highest morbidity in infants younger than six months [4]. Severe RSV disease may require advanced respiratory support and occurs disproportionately in preterm infants and children with cardiopulmonary comorbidities or immunodeficiency [5]. These acute clinical manifestations are accompanied by broader health-system and long-term consequences. Beyond its clinical impact, RSV causes a substantial global economic burden, with high inpatient and outpatient treatment costs estimated at approximately EUR 4.8 billion worldwide in 2017, largely driven by severe disease and hospitalizations [6]. In addition, RSV infections in early childhood, particularly severe cases requiring hospitalization, are associated with an increased risk of allergic sensitization [7], respiratory morbidity, recurrent wheezing, and asthma later in life [8]; however, this association diminishes with increasing age [9]. The relationship between RSV and atopy remains incompletely understood, as severe RSV infections are more frequently observed in conjunction with allergic sensitization, but a definitive causal link has not been established, and pre-existing airway vulnerability cannot be excluded [10].

The COVID-19 pandemic profoundly disrupted typical RSV circulation patterns. Strict non-pharmaceutical interventions (NPIs) led to a reduction in RSV activity in 2020/21, followed by atypically early and sometimes intensified seasons in 2021/22 and 2022/23 [5,11]. These post-NPI seasons were accompanied by shifts toward older age groups and raised concerns regarding an “immunity gap” in young children [12]. Since 2023/24, the introduction of mandatory national laboratory reporting in Germany has improved surveillance of RSV circulation and seasonal onset [13], though notification data alone do not capture clinical severity or intensive care burden. Simultaneously, RSV prevention has entered a transformative era. Long-acting monoclonal antibodies such as Nirsevimab (Sanofi-Aventis, Frankfurt, Germany) [14] and maternal vaccination with RSVpreF (Pfizer, Berlin, Germany) [15] have emerged as important tools to protect young infants from severe RSV disease [16,17]. In mid-2024, the German Standing Committee on Vaccination (STIKO) recommended universal Nirsevimab prophylaxis for all infants in their first RSV season, with implementation beginning before the 2024/25 season [14]. Early real-world data from other countries indicate substantial reductions in RSV-associated hospitalizations following high Nirsevimab coverage, although effects on intensive care admissions and age distributions have varied [18,19]. Robust German clinical data spanning the transition from the pandemic era to universal infant prophylaxis are still limited. Existing German reports have primarily addressed either population-level notification data or single-center hospital cohorts [20]. Few studies have linked epidemiological trends to detailed severity metrics such as length of stay, PICU admission, or respiratory support [21]. Moreover, there is little systematic analysis of pediatric emergency department (ED) burden, defined as the volume and characteristics of RSV-associated presentations requiring emergency evaluation [22]. Because RSV testing in EDs is selective and many mildly symptomatic children remain untested, ED data reflect service utilization rather than true population incidence [23]. Despite this limitation, ED burden provides essential insight into real-world healthcare demand, patterns of care-seeking behavior, and the capacity needs of pediatric emergency systems—information that is critical for anticipating seasonal strain and designing future prospective surveillance frameworks [24].

In the 2024/25 RSV-season (weeks 40/2024–20/2025), 67,892 RSV cases were reported in Germany, representing a moderate 17% increase compared with the previous season, alongside 118 reported RSV outbreaks, predominantly in daycare settings, long-term care facilities, and hospitals. Despite these higher overall notification numbers, RSV disease burden among infants declined markedly: RSV incidence in children < 1 year of age decreased by more than 50% compared with the prior season, accompanied by a clear reduction in RSV-associated hospitalizations. As infants are typically cared for outside of community settings, this decline is unlikely to be explained by outbreak dynamics alone and may represent an epidemiologic signal temporally associated with universal Nirsevimab prophylaxis on RSV-associated disease burden in the most vulnerable age group [25].

However, national notification data alone cannot capture clinical severity, care-setting distribution, or immunization-linked outcomes at the patient level. We combined data from two complementary pediatric centers in Bavaria. The University Children’s Hospital Augsburg provided detailed ICD-based epidemiological data on RSV-coded hospitalizations across three consecutive seasons, including granular age distributions and the first season of universal infant Nirsevimab implementation. The University Children’s Hospital Erlangen contributed a six-season cohort capturing inpatient and ED presentations, including hospital length of stay and PICU utilization, spanning the pre-pandemic period, the SARS-CoV-2 pandemic, and the immediate post-implementation season. Together, these datasets enabled a two-center, real-world assessment of RSV-associated healthcare burden—encompassing both inpatient care and ED burden—across a period of profound epidemiological and preventive change.

Therefore, the primary aim of this retrospective two-center cohort study was to descriptively characterize RSV-associated healthcare utilization before and after the introduction of universal immunoprophylaxis, without the intention to estimate vaccine effectiveness. Specifically, we aimed to i) describe temporal patterns and RSV-associated healthcare burden across six seasons (2019/20–2024/25), including changes during the COVID-19 pandemic and the first season following universal Nirsevimab prophylaxis; ii) characterize clinical severity among hospitalized RSV cases, including hospital length of stay and PICU admissions, and assess differences between centers; and iii) explore early real-world signals of shifts in age distribution and healthcare burden following Nirsevimab implementation, while explicitly avoiding causal inference. Findings were contextualized using national RSV surveillance data from Germany. This two-center approach provides, to our knowledge, the first combined assessment from Germany linking RSV epidemiology, ED burden, inpatient clinical severity, and early universal prophylaxis in the post-pandemic era.

## 2. Materials and Methods

### 2.1. Study Design and Setting

The primary aim of this retrospective, two-center observational study was to descriptively characterize RSV-associated healthcare utilization before and after the introduction of universal immunoprophylaxis, not to estimate vaccine effectiveness. The study was conducted at two tertiary-care pediatric hospitals in Bavaria, Germany: the University Children’s Hospital Augsburg (Swabia) and the University Children’s Hospital Erlangen (Franconia). The centers lie within comparable epidemiologic catchment areas and applied harmonized definitions for RSV seasons and case identification. The study aimed to integrate complementary datasets: Augsburg contributed detailed inpatient-level clinical and immunization data for three consecutive RSV seasons (2022/23–2024/25), whereas Erlangen provided a six-season dataset (2019/20–2024/25) encompassing both inpatient and ED encounters to characterize healthcare utilization patterns and age distributions across the pre-pandemic, pandemic, and post-pandemic periods. Given differences in data structures, diagnostic coding, and variable availability, analyses from the two centers were conducted separately and synthesized descriptively. Individual immunization status and detailed severity metrics were available only for Augsburg; accordingly, cross-site comparisons are descriptive and should be interpreted with caution. Maternal RSV vaccination was not routinely implemented in Germany during the study period and therefore did not contribute to population-level protection in this cohort.

### 2.2. Case Identification and Data Sources

#### 2.2.1. Augsburg

All pediatric admissions were extracted from the institutional electronic health record (ORBIS, Dedalus HealthCare GmbH, Bonn, Germany). RSV diagnoses were classified as primary diagnoses (PD), indicating RSV-attributable hospitalizations, and secondary diagnoses (SD), reflecting incidental RSV detection in hospitalizations for other primary conditions. RSV hospitalizations were identified using ICD-10 codes for primary diagnosis (PD): J12.1 (RSV pneumonia), J20.5 (RSV bronchitis), and J21.0 (RSV bronchiolitis). Secondary RSV diagnoses (SD) were defined as these codes in non-primary positions or B97.4 (“RSV as the cause of diseases classified elsewhere”). All children aged 0–17 years were eligible. Extracted variables included demographics, ICD-10 diagnoses, hospitalization dates, length of stay (LOS), PICU admission and duration of stay in days, and detailed respiratory support variables.

#### 2.2.2. Erlangen

RSV-positive encounters in inpatient wards and the ED were identified from fully anonymized line lists for each RSV season. All included encounters were confirmed positive by PCR and negative for SARS-CoV-2 and Influenza. Extracted variables included age (days and years), admission and discharge timestamps, LOS, PICU days, mortality, and ICD-10 primary diagnosis. ED and inpatient encounters were stored separately to distinguish service utilization (ED burden) from inpatient disease burden. Across seasons, Erlangen contributed inpatient encounters and ED-only RSV presentations. Because ED testing is selective, ED encounters were interpreted as healthcare utilization signals rather than indicators of population-level RSV incidence.

Because Nirsevimab became a novel standard of care in 2024/25, immunization status for infants < 1 year was extracted from neonatal charts, pediatric admission notes, and pharmacy administration logs in both centers.

### 2.3. RSV Season Definitions

RSV seasons were labeled according to regional epidemiologic practice:

- Erlangen: 2019/20–2024/25
- Augsburg: 2022/23–2024/25

For Augsburg, temporal analyses of season onset included all RSV admissions from September–April; all burden and severity analyses were restricted to October–March, consistent with national surveillance definitions.

### 2.4. Rationale for Age Stratification (< 1 Year and < 2 Years)

Age-based analyses focused on < 1 year and < 2 years for the following reasons:

1. Infants < 1 year: This group carries the highest risk for severe RSV, accounts for most PICU admissions, and is the primary target population for universal Nirsevimab prophylaxis. The < 1-year stratum allows evaluation of early real-world immunization effects and age-shifts in disease burden.
2. Children < 2 years: This stratum captures the population most susceptible to bronchiolitis and severe lower respiratory tract infection. Severity analyses (LOS, PICU burden, ventilatory support) were standardized to children < 2 years to support harmonized comparisons.
3. Children ≥ 2 years served as a comparator population with historically low risk for severe RSV disease.

Age was analyzed using both continuous values (days) and categorical groupings to enable harmonized analyses across datasets.

### 2.5. Assessment of Immunization Status and Timing (Augsburg)

For all infants < 1 year hospitalized in 2024/25, immunization status (Nirsevimab: yes/no/unknown) and age at admission were manually verified. During the RSV season, Nirsevimab administration in Augsburg was routinely offered in the context of the U2 well-child examination (day of life 3–10), which is universally scheduled for all newborns in Germany. While attendance at U2 is standard of care, immunization itself is voluntary and requires parental consent. Estimated immunization timing was calculated relative to date of birth. Infants were classified as protected at admission if Nirsevimab had been administered before hospitalization. In addition to patient-level verification, monthly birth-cohort immunization coverage was estimated by integrating neonatal ward immunization logs with institutional birth statistics.

### 2.6. Clinical Severity Outcomes

Clinical severity was assessed using hospital length of stay (LOS, days), PICU admission (yes/no), PICU length of stay (days), respiratory support modality (high-flow nasal cannula, non-invasive ventilation, invasive ventilation), and total ventilation hours where documented. These parameters were cross-validated against clinical documentation and procedural coding. Severity analyses were restricted to children younger than 2 years. An RSV admission was classified as severe if at least one predefined severity criterion was met (e.g., PICU admission, invasive ventilation, or vasoactive support). LOS was analyzed separately as a continuous outcome and was not included in the binary severity classification.

### 2.7. Statistical Analysis

All analyses were descriptive, reflecting the observational multicenter design, heterogeneous data availability across centers, and the availability of only a single post-implementation season following nirsevimab introduction. In the absence of harmonized individual-level exposure data across both centers, inferential or effectiveness analyses were not pursued. Continuous variables were summarized using medians and interquartile ranges (IQR), and categorical variables using absolute and relative frequencies. Age was analyzed both as a continuous measure (days) and in predefined categories (<1 year and <2 years) to enable harmonized comparisons across centers and to reflect clinically relevant risk strata for severe RSV disease.

Seasonal comparisons in Augsburg were descriptive and focused on changes in hospitalization burden, age distribution, and severity indicators (PICU admission, respiratory support, length of stay). Erlangen data were likewise summarized descriptively, with inpatient and emergency department (ED) encounters analyzed separately to distinguish inpatient burden from healthcare utilization patterns.

No formal hypothesis testing or multivariable modeling was undertaken. This approach was chosen because only a single season following the introduction of universal Nirsevimab prophylaxis was available, which precludes meaningful inference regarding the effectiveness of Nirsevimab prophylaxis or temporal trends. In addition, pandemic-associated disruptions in RSV circulation render traditional between-season statistical comparisons inappropriate. Finally, differences in case ascertainment and data structure between the two centers did not permit pooled estimates or direct comparative testing.

Therefore, numerical findings are reported as descriptive estimates intended to characterize healthcare burden, age distribution, and severity patterns across centers and seasons rather than to support inferential statistical conclusions.

All analyses were performed using GraphPad Prism 10.4.1 (GraphPad Software LLC, Boston, MA, USA, www.graphpad.com), with additional graphical output generated using MS Excel (Microsoft, Redmond, WA, USA).

## 3. Results

### 3.1. Overall RSV Burden Across Two Centers

**Figure 1:**
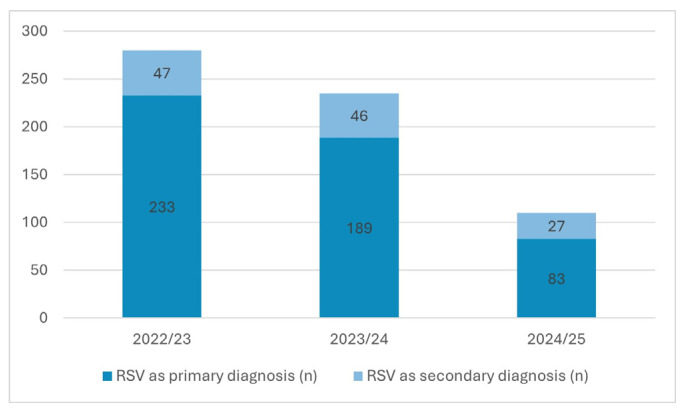
Seasonal RSV burden in Augsburg across three consecutive seasons (October–March), showing the number of inpatient hospitalizations/contacts with RSV as a primary diagnosis (PD) and the number of inpatient hospitalizations/contacts with RSV coded as a secondary diagnosis (SD). Primary RSV hospitalizations declined substantially in 2024/25 following the introduction of universal infant immunoprophylaxis, whereas SD encounters remained relatively stable, reflecting ongoing detection of RSV in children admitted for other primary conditions.

Across the two participating pediatric centers, RSV activity showed substantial temporal variability. Augsburg recorded *n* = 233, 189, and 83 hospitalizations with RSV as the primary diagnosis in the 2022/23, 2023/24, and 2024/25 seasons (Table 1), respectively, accompanied by *n* = 47, 46, and 27 RSV-associated secondary diagnoses (Table 2). A comprehensive list of ICD-coded primary diagnoses (PDs) for encounters with RSV as a secondary diagnosis (SDs) is available in Supplementary Table S1. Primary RSV hospitalizations declined substantially in 2024/25, coinciding with the introduction of universal infant immunoprophylaxis, whereas SD encounters remained relatively stable, reflecting ongoing detection of RSV in children admitted for other primary conditions (Figure 1).

Erlangen contributed six seasons of PCR-confirmed RSV encounters, demonstrating 45 cases in 2019/20, an absence of RSV in 2020/21, and a subsequent resurgence with *n* = 76, 104, 100, and 45 encounters in 2021/22 through 2024/25. Of these, 34–89 cases per season required inpatient admission, while 10–32 encounters per season represented RSV-positive ED presentations without hospitalization. The suppression of RSV circulation in 2020/21 reflects the profound impact of pandemic-related non-pharmaceutical interventions (Table 3).

### 3.2. Seasonal Timing of RSV Activity

RSV onset varied across years in both centers. In Augsburg, the first RSV hospitalization occurred in mid-September during the 2022/23 season, in November during 2023/24, and in mid-December during 2024/25. Erlangen demonstrated a similar degree of temporal variability, including a complete absence of RSV-positive encounters in 2020/21. These data illustrate the substantial year-to-year variation in the timing of RSV activity observed across the study period.

### 3.3. Age Distribution Across Centers

**Figure 2:**
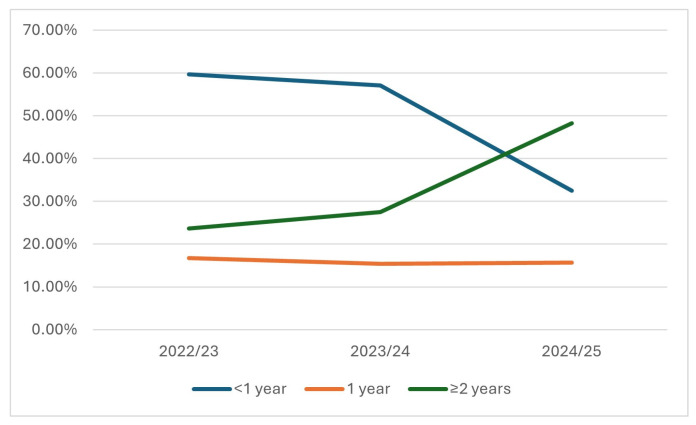
Age distribution of hospitalized RSV cases in Augsburg across three consecutive seasons (October–March). The figure shows the season-specific proportion of RSV hospitalizations (primary RSV diagnosis in Augsburg) occurring in infants < 1 year (blue), 1-year-old children (orange), and children ≥ 2 years (green), with each proportion calculated using the total number of RSV hospitalizations in the respective season as the denominator. A marked decline in the proportion of <1-year admissions was observed in 2024/25 (32.5%) compared with pre-immunization seasons (~60%), accompanied by a corresponding increase in hospitalizations among children ≥ 2 years. These shifts coincide with the first season of universal Nirsevimab implementation. Percentages may not sum to 100% due to rounding.

Despite center-specific differences in data structure and case ascertainment, RSV encounters consistently clustered in early infancy. In Augsburg, infants < 1 year accounted for 59.7% and 57.1% of inpatient cases in the two pre-immunization seasons but only 32.5% in 2024/25, representing a pronounced shift temporally coincident with the first season of Nirsevimab rollout (Table 3, Figure 2). Secondary-diagnosis RSV cases showed a similar redistribution toward older ages in 2024/25 (Table 4). The proportion of male patients was consistent across the 2022/23–2024/25 seasons, varying between 49.2% and 55.4% (Table 4).

In Erlangen, hospitalized patients remained predominantly young across all seasons, with 52–62% <1 year and 70–80% <2 years, reflecting typical RSV epidemiology. ED encounters demonstrated a broader age spectrum, with <1-year-olds representing 33–55% depending on the season (Table 3). Together, the two datasets indicate that RSV burden remains concentrated in infancy but that the relative contribution of the youngest infants to hospital burden decreased substantially in Augsburg following the introduction of universal infant prophylaxis.

**Table 4:** Distribution of RSV-related primary (PD) and secondary diagnosis (SD) by ICD category and age group across seasons in Augsburg. SD encounters were grouped into four clinically relevant ICD clusters (J12.1 viral pneumonia, J20.5 acute bronchitis/bronchiolitis, J21.0 bronchiolitis, B97.4 RSV as cause of diseases elsewhere). Age distribution is shown for infants < 1 year, 1-year-olds, and children ≥ 2 years.

Season	Total RSV PD	% Male (PD)	Infants < 1 Years, *n* (%)	Children < 2 Years, *n* (%)	Total SD	Infants < 1 Years with SD (%)
2022/23	233	55.4%	139 (59.7%)	178 (76.4%)	47	29.8%
2023/24	189	49.2%	108 (57.1%)	137 (72.5%)	46	32.6%
2024/25	83	54.2%	27 (32.5%)	40 (48.2%)	27	7.4%

### 3.4. ED Burden and Care-Setting Distribution

Erlangen’s dual-setting dataset provides unique insight into healthcare utilization patterns. The proportion of RSV encounters managed exclusively in the ED increased steadily over time—from 24% in 2019/20 to 32% and 44% in 2023/24 and 2024/25, respectively—despite declining total encounter numbers. This rising ED burden underscores that ED-based RSV metrics primarily reflect real-world testing behavior and care-seeking patterns rather than true infection incidence, a distinction critical for interpreting surveillance data in clinical and public health contexts.

### 3.5. Clinical Severity Among Hospitalized Children

**Figure 3:**
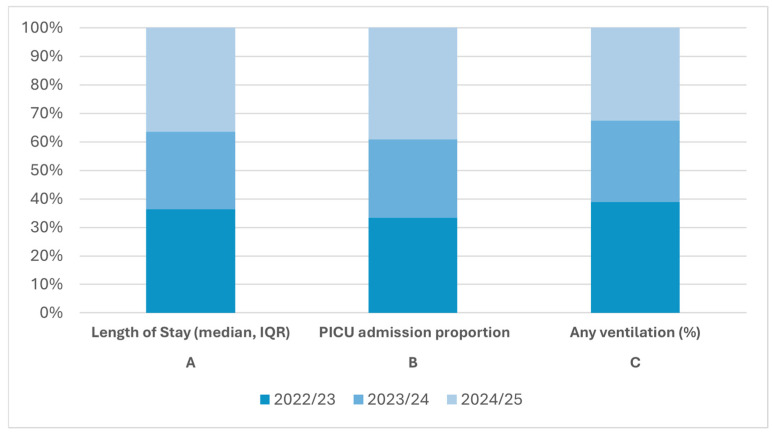
Clinical severity of RSV infection in children < 2 years across three RSV seasons (Augsburg, October–March). Each panel displays seasons as 100% stacked bars to illustrate the relative proportion of severity outcomes per season; absolute values are provided in the caption for clarity. Panel A shows median length of stay (LOS) with interquartile ranges (IQR; 2022/23: 4 days, 2023/24: 3 days, 2024/25: 4 days). Panel B shows the proportion of hospitalized children requiring PICU admission (10.7%, 8.8%, and 12.5%, respectively). Panel C shows the proportion receiving any form of ventilatory support (9.0%, 6.6%, and 7.5%, respectively). Using proportional visualization highlights that LOS, PICU utilization, and ventilatory support rates remained stable across seasons, indicating no increase in clinical severity after the introduction of universal infant immunoprophylaxis.

Across centers, there was no evidence of increasing clinical severity over time. In Augsburg, among children < 2 years, median length of stay remained 3–4 days across all seasons, PICU admission rates ranged from 8.8% to 12.5%, and ventilatory support was required in 6.6–9.0% of cases; PICU days per 100 ward-days were stable. Erlangen’s inpatient cohort showed similarly consistent severity profiles, with median stays of 3–6 days and PICU admission rates of 4–12% across most seasons. Although a higher proportion required PICU care in 2024/25, ICU stays were short, indicating brief escalation rather than sustained severity. Collectively, these findings suggest that hospitalization thresholds and disease severity remained unchanged despite fluctuations in burden and age distribution (Figure 3).

### 3.6. Immunization-Specific Outcomes in Infants < 1 Year (Augsburg, 2024/25)

**Figure 4:**
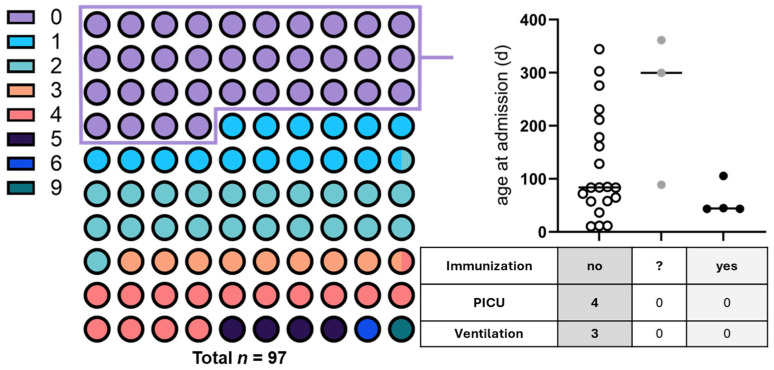
Age distribution of RSV-related hospitalizations in Augsburg during the 2024/25 season and severity outcomes in infants < 1 year of age. The left panel displays the age distribution of all children hospitalized with RSV infection in Augsburg during the 2024/25 season (*n* = 97 hospitalizations), color-coded by age group (0–9). Color code “0” (purple) represents infants younger < 1 year of age, while increasing numbers indicate older pediatric age groups. The subgroup of infants < 1 year (*n* = 27 hospitalizations, purple) is analyzed in detail in the right panel, which shows the distribution of hospitalized infants < 1 year by immunization status, along with associated severity outcomes. The table summarizes PICU admissions and the need for ventilatory support. Dot fill denotes immunization status (open = not immunized (no); grey = unknown (?); solid = immunized (yes)). Notably, in this cohort, all severe outcomes, including four PICU admissions and three cases requiring ventilatory support, among infants < 1 year occurred in unimmunized infants; however, the number of immunized cases was small. No immunized infant required PICU care or respiratory support.

A detailed assessment of infants < 1 year in the first Nirsevimab season revealed a striking divergence in clinical outcomes by immunization status. Among 27 hospitalized infants, four had received Nirsevimab, twenty were unimmunized, and three had unknown status (Figure 4).

Notably, in this cohort, all severe RSV cases among infants < 1 year occurred in unimmunized infants; however, the number of immunized cases was small. In contrast, none of the immunized infants required respiratory support or intensive care, despite substantial medical complexity in three cases: (i) an infant with trisomy 21 and associated pulmonary hypertension, congenital nephrotic syndrome, and dystrophy; (ii) an extremely preterm infant (24 + 4 weeks, 407 g birth weight) with severe bronchopulmonary dysplasia, obstructive left ventricular outflow tract disease, and a history of NEC IIb with stoma; and (iii) a former 31-week preterm infant recently recovering from influenza B infection. Diagnostic patterns among immunized infants resembled those of unimmunized infants and were dominated by bronchiolitis. Estimated intervals between immunization and hospitalization placed all immunized infants well within the expected window of protection.

### 3.7. Inpatient Immunization Uptake (Augsburg and Erlangen Birth Cohort, 2024/25)

To contextualize early implementation of the national recommendation, inpatient Nirsevimab administration at birth was assessed at both study centers (Supplementary Tables S2 and S3). Among all live-born infants delivered at the maternity wards between October 2024 and March 2025 (Augsburg: *n* = 1204; Erlangen: *n* = 1156), 800 infants in Augsburg (66.4%) and 675 infants in Erlangen (58.4%) received Nirsevimab prior to discharge. These data capture inpatient administration at birth and should not be interpreted as population-wide regional immunization coverage.

The observed proportions therefore represent real-world uptake of the U2-based immunization strategy during the first season of universal prophylaxis. Among infants < 1 year of age in Augsburg who were subsequently hospitalized with RSV during the same period, 14.8% (4/27) had received Nirsevimab prior to admission, representing the proportion of immunized infants among hospitalized cases. Three additional otherwise healthy immunized infants were hospitalized in April outside the analytic window.

## 4. Discussion

### 4.1. Summary of Main Findings

Across two pediatric centers and six observation seasons, we identified several converging signals characterizing RSV epidemiology during the transition from the pandemic era to the first season of universal infant immunoprophylaxis. In Augsburg, RSV hospitalizations with RSV as the primary diagnosis declined markedly in 2024/25, particularly among infants < 1 year—the group historically at highest risk of severe disease—while markers of clinical severity in hospitalized children < 2 years (PICU admission, ventilatory support, length of stay) remained unchanged. A gradual temporal shift toward later RSV season onset was evident, with the first hospitalization in 2024/25 occurring only in mid-December. Notably, in this cohort, all severe RSV cases among infants < 1 year occurred in unimmunized infants; however, the number of immunized cases was small. None of the immunized infants—including those with substantial medical complexity—required PICU care or respiratory support.

During the same period, inpatient Nirsevimab uptake reached 66.4% of newborns before discharge, despite only moderate pre-season parental acceptance. Erlangen’s six-season dataset provided essential epidemiologic context, demonstrating stable inpatient severity, marked variability in seasonal timing, and a rising proportion of RSV-positive encounters managed exclusively in the emergency department (ED). Together, these findings provide a coherent two-center assessment of evolving RSV dynamics during a period of major epidemiologic and preventive change.

### 4.2. Interpretation in Context of Existing Evidence

Our findings descriptively document a coincident reduction in RSV-related hospitalization burden following the introduction of universal Nirsevimab prophylaxis among young infants. Similar temporal patterns have been reported in national surveillance data from Germany [20] and other countries implementing broad immunoprophylaxis programs [15,16,24,25,26,27]. These findings should be viewed as hypothesis-generating real-world observations rather than causal or effectiveness estimates.

Interestingly, data from Italy suggest that not only coverage but also the timing of Nirsevimab administration is relevant. Regions that implemented prophylaxis early—before or at the beginning of the RSV season—experienced substantially lower rates of RSV-associated hospitalizations and milder disease courses in infants compared with regions where rollout was delayed [28]. Implementation studies from Spain further indicate that delays in immunization—particularly in infants not hospitalized at birth—may attenuate early-season protection, underscoring the importance of timely program rollout [18].

The stable severity indicators across seasons—both in Augsburg and Erlangen—support the interpretation that fewer infants developed severe RSV disease, rather than a shift in clinical thresholds for hospital or PICU admission. The markedly delayed onset of the 2024/25 season is particularly noteworthy [25]. Similar shifts have been described in regions with high Nirsevimab uptake, raising the hypothesis that early protection in the youngest birth cohort dampens early-season transmission [29]. While causal inference is not possible, the temporal alignment between strong early inpatient uptake and delayed RSV onset supports this theory and merits evaluation in broader surveillance networks [18,29].

### 4.3. Seasonal Timing and Potential Drivers of Variation

The variability in season onset across our study period—from an unusually early start in 2022/23 [5] to a mid-season onset in 2023/24 [13] and a significantly delayed onset in 2024/25—is consistent with national reports of unstable RSV circulation following the COVID-19 pandemic [25]. The delayed 2024/25 onset occurred during the first season of universal infant Nirsevimab availability and may reflect altered transmission dynamics in a population with substantial early coverage. Future national-level analyses will be required to determine whether this pattern generalizes beyond our region.

### 4.4. Immunization-Specific Severity Patterns in Infants

The immunization-specific subgroup analysis provides the clearest and most clinically relevant signal in this study. In our cohort, none of the immunized infants required respiratory support or PICU admission, despite the presence of substantial pulmonary and systemic comorbidities, including former extreme prematurity with bronchopulmonary dysplasia, trisomy 21 with pulmonary hypertension and congenital nephrotic syndrome, and recent influenza infection. In contrast, all severe RSV cases occurred exclusively among unimmunized infants.

This finding is of particular clinical relevance, as pre-existing conditions and comorbidities—especially multiple underlying diseases and congenital cardiac anomalies—are well-established risk factors for severe RSV disease and mortality, and nosocomial RSV acquisition represents an additional major mortality risk in this population [30]. Among the small number of immunized infants hospitalized with RSV, no severe outcomes were observed; however, given the very limited case number, this finding may reflect chance rather than a true protective effect.

The similar diagnostic spectrum observed among immunized and unimmunized infants indicates that immunoprophylaxis does not appear to alter the clinical phenotype of RSV infection in hospitalized cases [31]. This observation is biologically compatible with the known mechanism of action of Nirsevimab and with efficacy profiles reported in pivotal clinical trials [32,33], as well as with available safety data in infants with underlying cardiac or pulmonary disease [34]. These observations are descriptive in nature and should be interpreted cautiously, without inference regarding effectiveness or causality, particularly in light of the observational design and limited post-implementation follow-up.

### 4.5. Parental Attitudes and Real-World Uptake

Understanding these clinical findings requires contextualization within early adoption dynamics and implementation maturity. A pre-season (2024/25) survey conducted in the participating maternity units showed that although 78% of parents were aware of RSV, only 53% supported immunization on day 3 of life, reflecting early ambiguity regarding the timing of the final STIKO recommendation, the setting of administration, and reimbursement pathways [35]. Implementation success therefore also reflects broader system-level factors. While initial logistical uncertainty posed barriers for both parents and clinicians during the early adoption phase, coordinated communication by health authorities and the establishment of integrated workflows between maternity wards and community pediatricians—as reflected in our parental survey and subsequent real-world uptake data [35]—appear to have facilitated a transition toward operational maturity over the course of the season, resulting in moderate to high overall inpatient coverage (58.4% in Erlangen and 66.4% in Augsburg). In our hospitals, Nirsevimab administration was operationally aligned with the routinely scheduled U2 postnatal assessment rather than at delivery, embedding immunization within established maternity–pediatric care pathways and supporting its uptake during the early implementation phase. Although our study cannot distinguish inpatient from outpatient administration, and individual immunization status at RSV admission was available only in Augsburg, severe RSV disease occurred exclusively among unimmunized infants, suggesting that the achieved overall coverage was compatible with the observed reduction in severe RSV disease at the population level, although causal inference is not possible.

### 4.6. RSV as a Secondary Diagnosis: Epidemiologic and Clinical Relevance

Across seasons in Augsburg, the number of hospitalizations with RSV coded as a secondary diagnosis remained relatively stable, even as hospitalizations with RSV as the primary diagnosis declined sharply in 2024/25. This distinction is critical for interpretation, as RSV listed as a secondary diagnosis typically reflects incidental viral detection in children admitted for unrelated conditions, such as non-RSV pneumonia, febrile illness, or surgical and cardiac disorders, and therefore does not represent RSV-attributable disease burden. The stability of secondary RSV diagnoses across seasons thus provides an internal consistency check, supporting the interpretation that the marked decline in primary RSV hospitalizations reflects a genuine reduction in RSV-driven clinical disease rather than shifts in testing practices, diagnostic coding, or admission thresholds.

### 4.7. Insights from ED Utilization and the Multicenter Context

Erlangen’s dual-setting dataset shows a rising proportion of RSV-positive encounters managed exclusively in the ED. Because RSV testing in ED settings is selective and symptom-driven, counts of RSV-related ED encounters primarily capture patterns of healthcare utilization rather than true community-level incidence. This highlights the importance of interpreting ED-based RSV metrics within the context of testing practices and care-seeking behavior. The stability of inpatient severity in Erlangen reinforces that the decline in Augsburg’s primary RSV hospitalizations is unlikely to be driven by changes in admission thresholds.

The multicenter approach thus provides a multidimensional perspective: Augsburg contributed granular severity and immunization-linked insights, while Erlangen provided the broader epidemiologic and care-setting variability needed to contextualize these findings. Together, these perspectives provide a robust characterization of RSV epidemiology across pre-pandemic, pandemic, and early post-implementation phases.

### 4.8. Strengths and Limitations

Strengths include detailed patient-level severity data, the ability to directly compare outcomes in immunized and unimmunized infants, and the integration of inpatient and ED encounters across two complementary centers. Few early real-world evaluations of Nirsevimab have been able to link immunization status to clinically meaningful severity markers. With respect to data availability across centers, immunization-linked analyses of clinical severity were available only from Augsburg, whereas Erlangen primarily contributed epidemiologic and care-setting context rather than direct immunization outcome data. Although length of stay, PICU admission, and ventilatory support are clinically meaningful severity indicators, more granular physiologic measures (e.g., oxygen requirement trajectories or standardized severity scores) were not uniformly available across centers. In addition, as outpatient data regarding Nirsevimab administration were not available, overall regional coverage beyond inpatient birth cohorts could not be assessed. Moreover, while reduced RSV-associated admissions likely translated into relief of pediatric ward and intensive care capacity during peak season, center-wide bed occupancy data were not available for systematic analysis and could not be formally assessed in this study. Further limitations include incomplete documentation of exact immunization dates, which required estimation based on preventive care schedules; inability to distinguish inpatient from outpatient administration; variable granularity in respiratory support documentation; and potential misclassification of secondary RSV diagnoses, though seasonal patterns were highly consistent. Furthermore, although parental attitudes and real-world outcomes derive from the same two centers, the datasets could not be linked at the individual level. Observed changes may in part reflect evolving RSV seasonality and healthcare-seeking behavior following the COVID-19 pandemic, independent of immunoprophylaxis. These factors cannot be fully disentangled in a descriptive real-world design. Finally, Augsburg’s burden analysis reflects a single center, though interpretation is strengthened substantially by triangulation with Erlangen’s six-season dataset.

## 5. Conclusions

This two-center study provides early real-world descriptive data coinciding with a marked reduction in RSV-related hospitalizations following the introduction of universal infant immunoprophylaxis with Nirsevimab. No severe RSV outcomes were observed among the small number of immunized infants, including those with significant comorbidities, although causal inference is not possible. Clinical severity among hospitalized children remained stable across seasons, supporting the interpretation that changes in hospitalization burden were not driven by shifts in admission practices. The observed delay in seasonal onset and changes in age distribution occurred during the first season of universal prophylaxis and warrant further investigation in larger surveillance datasets. Together, these findings highlight the value of integrating emergency department and inpatient data to characterize RSV-associated healthcare utilization during a period of substantial epidemiologic transition.

## Figures and Tables

**Table 1:** Primary RSV diagnoses (PD; all ages) by ICD-10 code and RSV season in Augsburg, restricted to the analytic window (October–March). Counts reflect hospitalizations coded with RSV as the PD; out-of-window cases (April) were excluded for consistency with all burden and severity analyses.

ICD Code (Primary Diagnosis)	2022/23	2023/24	2024/25	Total ^1^
J12.1 RSV pneumonia	41	51	23	115
J20.5 RSV bronchitis	81	39	32	152
J21.0 RSV bronchiolitis	113	101	28	242
Total RSV primary diagnoses (PD)	233	189	83	505

^1^ Outside the analytic window (Oct–Mar), additional RSV primary diagnoses (PDs) occurred in September 2022 (*n* = 2, both J12.1), April 2023 (*n* = 2, both J21.0). Fourteen April 2025 cases were excluded (J20.5 *n* = 7, J21.0 *n* = 3, J12.1 *n* = 4).

**Table 2:** RSV coded as a secondary diagnosis (SD; all ages) by ICD-10 category and RSV season in Augsburg, restricted to the analytic window (October–March).

ICD-Code (Secondary Diagnosis)	2022/23	2023/24	2024/25	Total ^1^
B97.4 RSV as causative agent	35	37	22	94
J12.1 RSV pneumonia (SD)	5	2	2	9
J20.5 RSV bronchitis (SD)	3	4	1	8
J21.0 RSV bronchiolitis (SD)	4	3	2	9
Total RSV secondary diagnoses (SD)	47	46	27	120

^1^ April 2025 secondary diagnoses (SD) add-on (i.e., outside main window): 4 additional patients with RSV as SD (H70.0, G40.9, J06.8, N10 as PDs).

**Table 3:** Age distribution of RSV-positive encounters across two pediatric centers (Augsburg inpatient; Erlangen inpatient and emergency department), stratified by RSV season. The table reports the proportions of children < 1 year, < 2 years, and ≥2 years among all RSV-positive encounters. Augsburg shows a pronounced reduction in <1-year-old hospitalizations in 2024/25, consistent with early effects of universal infant immunoprophylaxis, whereas Erlangen demonstrates more stable age distributions. As expected, ED encounters include a relatively older population, supporting the interpretation of RSV-related ED visits as indicators of service utilization rather than disease incidence. Legend: ED = emergency department. *n* denotes RSV-positive encounters (not unique patients).

Season	Center/Setting	*n*	% <1 Year	% <2 Years	% ≥2 Years
2019/20	Erlangen—Inpatient	34	55.9%	67.6%	32.4%
	Erlangen—ED	11	54.5%	63.6%	36.4%
2020/21	Erlangen—Inpatient	0	–	–	–
	Erlangen—ED	0	–	–	–
2021/22	Erlangen—Inpatient	66	62.1%	80.3%	19.7%
	Erlangen—ED	10	50.0%	90.0%	10.0%
2022/23	Augsburg—Inpatient	233	59.7%	76.4%	23.6%
	Erlangen—Inpatient	89	51.7%	69.7%	30.3%
	Erlangen—ED	15	33.3%	66.7%	33.3%
2023/24	Augsburg—Inpatient	189	57.1%	72.5%	27.5%
	Erlangen—Inpatient	68	61.8%	73.5%	26.5%
	Erlangen—ED	32	43.8%	68.8%	31.2%
2024/25	Augsburg—Inpatient	83	32.5%	48.2%	51.8%
	Erlangen—Inpatient	25	52.0%	72.0%	28.0%
	Erlangen—ED	20	35.0%	55.0%	45.0%

## Data Availability

The datasets generated and analyzed during the current study contain sensitive clinical information and cannot be made publicly available in order to protect patient privacy. Fully anonymized, aggregated data supporting the main findings of this study may be made available from the corresponding author upon reasonable request and subject to institutional and ethical regulations.

## Supplementary Materials

The following supporting information can be downloaded at: https://www.mdpi.com/article/10.3390/children13020173/s1, Table S1: Distribution of primary diagnoses among admissions with RSV coded as a secondary diagnosis (SD), grouped by ICD chapter. The table summarizes the primary diagnoses (PDs) for encounters in which RSV was recorded only as a secondary code, illustrating the underlying clinical reasons for hospitalization in this cohort; Table S2: Monthly Birth Cohort and Inpatient Nirsevimab Immunization Uptake (Augsburg, Oct 2024–Mar 2025); Table S3: Monthly Birth Cohort and Inpatient Nirsevimab Immunization Uptake (Erlangen, Oct 2024–Mar 2025).

## Abbreviations

The following abbreviations are used in this manuscript:

COVID-19	Coronavirus disease 2019
ED	Emergency department
EHR	Electronic health record
FAU	Friedrich-Alexander-Universität Erlangen-Nürnberg
GDPR	General Data Protection Regulation
ICD-10	International Classification of Diseases, 10th Revision
IfSG	Infektionsschutzgesetz (German Infection Protection Act)
IQR	Interquartile range
LOS	Length of stay
MS	Microsoft
NEC	Necrotizing enterocolitis
NICU	Neonatal intensive care unit
NPIs	Non-pharmaceutical interventions
ORBIS	ORBIS hospital information system (Dedalus)
PCR	Polymerase chain reaction
PD	Primary diagnosis
PICU	Pediatric intensive care unit
PPG	Photoplethysmography
RSV	Respiratory syncytial virus
RSVpreF	RSV prefusion F protein (maternal vaccine)
SARS-CoV-2	Severe acute respiratory syndrome coronavirus 2
SD	Secondary diagnosis
STIKO	Standing Committee on Vaccination (Germany)
