# Supplementary material for: Burden of RSV-Associated Inpatient Care and Emergency Service Utilization in Two German Pediatric Centers Across Six Seasons Including the First Nirsevimab Year
Source: Children (Basel). 2026 Jan 26;13(2):173. doi: 10.3390/children13020173 (PMC12939032; doi:10.3390/children13020173)
Supplement: Supplementary file 1 [file children-13-00173-s001.zip › children-4133721-supplementary.pdf]

Supplementary Table S1. Distribution of primary diagnoses among admissions with RSV coded as a secondary diagnosis (SD), grouped by ICD chapter. The table summarizes the primary diagnoses (PDs) for encounters in which RSV was recorded only as a secondary code, illustrating the underlying clinical reasons for hospitalization in this cohort.

ICD chapter group (primary diagnosis)	2022/23 (n=47)	2023/24 (n=46)	2024/25 (n=23)
Respiratory disease (non-RSV J-codes, e.g. URTI, croup)	15	27	13
Injury / poisoning (S, T)	8	4	0
Genitourinary disease (N)	6	0	0
Infectious & parasitic disease (A, B)	6	4	2
Nervous system (G)	3	1	2
Neoplasms / blood disorders (C, D)	2	3	3
Congenital malformations (Q)	2	1	0
Eye / ear disease (H)	2	1	0
Skin and subcutaneous tissue (L)	1	0	2
Digestive system (K)	1	1	0
Circulatory system (I)	0	1	1
Symptoms / ill-defined conditions (R)	1	3	0

Supplementary Table S2. Monthly Birth Cohort and Inpatient Nirsevimab Immunization Uptake (Augsburg, Oct 2024 - Mar 2025).

Month (2024/25)	Newborns	Inpatient immunized	Inpatient uptake (%)
October 2024	236	10	4.2%
November 2024	205	94	45.9%
December 2024	178	135	75.8%
January 2025	195	173	88.7%
February 2025	188	190	**>100%***
March 2025	202	198	98.0%
**Total (Oct–Mar)**	**1,204**	**800**	**66.4%**

* Uptake >100% reflects real-world clinical and documentation factors, including (i) immunization of infants born in previous months who remained hospitalized (e.g., preterm infants or prolonged NICU stays) and (ii) batch documentation delays, in which newborns delivered late in one month were recorded as immunized in the following month.

Supplementary Table S3. Monthly Birth Cohort and Inpatient Nirsevimab Immunization Uptake (Erlangen, Oct 2024 - Mar 2025).

Month (2024/25)	Newborns	Inpatient immunized	Inpatient uptake (%)
October 2024	218	22	7.68%
November 2024	188	95	50.53%
December 2024	166	127	76.51%
January 2025	182	151	82.97%
February 2025	193	146	75.65%
March 2025	209	134	64.11%
**Total (Oct–Mar)**	**1,156**	675	**58.39%**
